# Antithrombotic Therapy in Patients with Atrial Fibrillation Undergoing Percutaneous Coronary Intervention

**DOI:** 10.3390/jcm15187298

**Published:** 2026-09-19

**Authors:** Carmelo Raffo, Daniele Giacoppo, Davide Capodanno, Antonio Greco

**Affiliations:** Division of Cardiology, Azienda Ospedaliero-Universitaria “G. Rodolico–San Marco”, University of Catania, via Santa Sofia, 78, 95123 Catania, Italy

**Keywords:** acute coronary syndrome, atrial fibrillation, antithrombotic therapy, coronary artery disease, percutaneous coronary intervention

## Abstract

Antithrombotic therapy management in patients with atrial fibrillation (AF) undergoing percutaneous coronary intervention (PCI) remains challenging as it requires simultaneous protection from cardioembolism and coronary ischemic events while minimizing bleeding. The coexistence of these competing risks, along with their different temporal profiles, has driven a major shift in therapeutic strategies over the past decade. Randomized trials have consistently shown that direct oral anticoagulant (DOAC)-based dual antithrombotic therapy (DAT) reduces bleeding as compared with prolonged triple antithrombotic therapy (TAT), supporting early aspirin withdrawal in most patients. At the same time, pooled analyses suggest that this simplification may be accompanied by a small increase in ischemic risk, particularly during the first month after PCI, when the hazard of stent thrombosis is the highest. Contemporary guideline recommendations from Europe and North America are now broadly aligned in favoring a short periprocedural course of TAT followed by DAT with a DOAC and clopidogrel, then transitioning to oral anticoagulant monotherapy. However, important uncertainty remains for patients with very high ischemic or bleeding risk, including those with acute coronary syndromes, complex PCI, prior stent thrombosis, frailty, chronic kidney disease, previous bleeding, or active cancer. In these settings, treatment decisions cannot rely solely on trial-derived estimates or isolated risk scores, but require individualized, dynamic, and phase-specific assessments. This review critically appraises the biologic rationale, randomized evidence, and contemporary guideline recommendations for antithrombotic therapy in patients with AF undergoing PCI, focusing on areas not fully resolved by current guidelines, including modulation strategies, high-risk and underrepresented populations, tailored antithrombotic management, and emerging therapeutic directions.

## 1. Introduction

Atrial fibrillation (AF) is the most common sustained cardiac arrhythmia in clinical practice and a major global public health challenge, affecting more than 33 million people worldwide, with prevalence increasing with age [1]. AF is associated with a 4- to 5-fold higher risk of ischemic stroke and systemic embolism than in the general population, complications marked by high mortality and substantial residual disability that contribute materially to the global cardiovascular disease burden [2]. At the same time, coronary artery disease (CAD) remains the leading cause of morbidity and mortality worldwide [3,4]. Although AF and CAD are distinct clinical entities, they share major pathophysiological mechanisms and cardiovascular risk factors, including hypertension, diabetes mellitus and obesity, which help explain their frequent coexistence in clinical practice, with CAD affecting up to 40% of patients with AF [5,6].

Percutaneous coronary intervention (PCI) with stent implantation is a cornerstone of revascularization in CAD [7,8]. However, AF is present in approximately 10% of patients undergoing PCI, introducing substantial therapeutic challenges [9,10]. In this context, the central clinical challenge is the simultaneous prevention of distinct thrombotic mechanisms. In particular, AF-related cardioembolic risk requires oral anticoagulation (OAC), whereas prevention of stent-related ischemic events relies on dual antiplatelet therapy (DAPT) with aspirin and a P2Y_12_ receptor inhibitor [11,12,13]. The combination of OAC with DAPT, commonly referred to as triple antithrombotic therapy (TAT), provides comprehensive antithrombotic protection but is limited by a substantial increase in bleeding risk [14]. In particular, adding a single antiplatelet agent to OAC increases bleeding risk by 20% to 60%, whereas TAT increases this risk 2- to 3-fold, limiting its sustainability beyond the short term [2].

Despite extensive randomized evidence and evolving guideline recommendations, clinical practice remains quite heterogeneous [9]. Although contemporary guidelines define the general composition and duration of antithrombotic therapy, several clinically relevant uncertainties persist regarding the optimal timing of aspirin withdrawal, the intensity and duration of combination therapy in patients with competing ischemic and bleeding risks, the role of potent P2Y_12_ inhibitors, and the timing of transition to OAC monotherapy, particularly in patients at very high thrombotic or bleeding risk [15].

Accordingly, this review critically appraises contemporary evidence on antithrombotic strategies in patients with AF undergoing PCI, focusing on areas not fully resolved by current guidelines, including de-escalation strategies, high-risk and underrepresented populations, and tailored antithrombotic management.

## 2. The Ischemia–Bleeding Balance

In patients with AF who undergo PCI, the prevention of thrombotic complications requires simultaneous modulation of mechanistically distinct pathways. Stent thrombosis is predominantly platelet-mediated and reflects the exposure of circulating blood to a thrombogenic metallic scaffold in the setting of procedure-related endothelial disruption [16,17]. In acute coronary syndrome (ACS), plaque rupture or erosion similarly promotes the formation of platelet-rich thrombi [18]. By contrast, AF promotes thrombus formation through blood stasis and loss of effective atrial contractility, particularly within the left atrial appendage, leading to fibrin- and erythrocyte-rich thrombi driven by activation of the coagulation cascade [19,20]. This biologic divergence provides the rationale for combining OAC with antiplatelet therapy [14].

The competing and intertwined risks of ischemic events and bleeding are characterized by different temporal patterns [21]. After PCI, ischemic risk is characterized by marked temporal clustering. Among patients with ST-segment elevation myocardial infarction (STEMI) undergoing primary PCI, ischemic events occur most frequently during the acute and subacute phases, peaking within the first 24 h, remaining elevated during the subsequent 30 days, and progressively declining thereafter in parallel with vascular healing and stent endothelialization [22]. Although early mortality is higher than in stable CAD, long-term event rates tend to converge over time [23]. Residual ischemic risk is further modified by clinical, anatomic, and procedural features, including ACS presentation, prior stent thrombosis, multivessel or left main/proximal left anterior descending artery disease, complex PCI, diabetes, chronic kidney disease, and peripheral artery disease, which help define the period during which intensified antiplatelet therapy is most likely to provide net benefit [24,25]. However, as the risk of stent-related thrombosis recedes, bleeding assumes increasing clinical importance.

Bleeding associated with antithrombotic therapy follows a different course. While the risk of stent thrombosis attenuates after the initial weeks, bleeding risk remains substantial and accumulates over time [17,26]. After primary PCI for STEMI, bleeding events cluster early, largely as a consequence of vascular access and treatment intensity [22]. However, rehospitalizations for spontaneous hemorrhage, including gastrointestinal bleeding, continue to accrue beyond the acute phase [27,28]. DAPT maintains an elevated hemorrhagic risk for several months, and among patients at high bleeding risk (HBR), major events may occur at relatively constant rates beyond the first year [17]. In this evolving equilibrium, the requirement for long-term OAC in AF adds further complexity.

**Figure 1:**
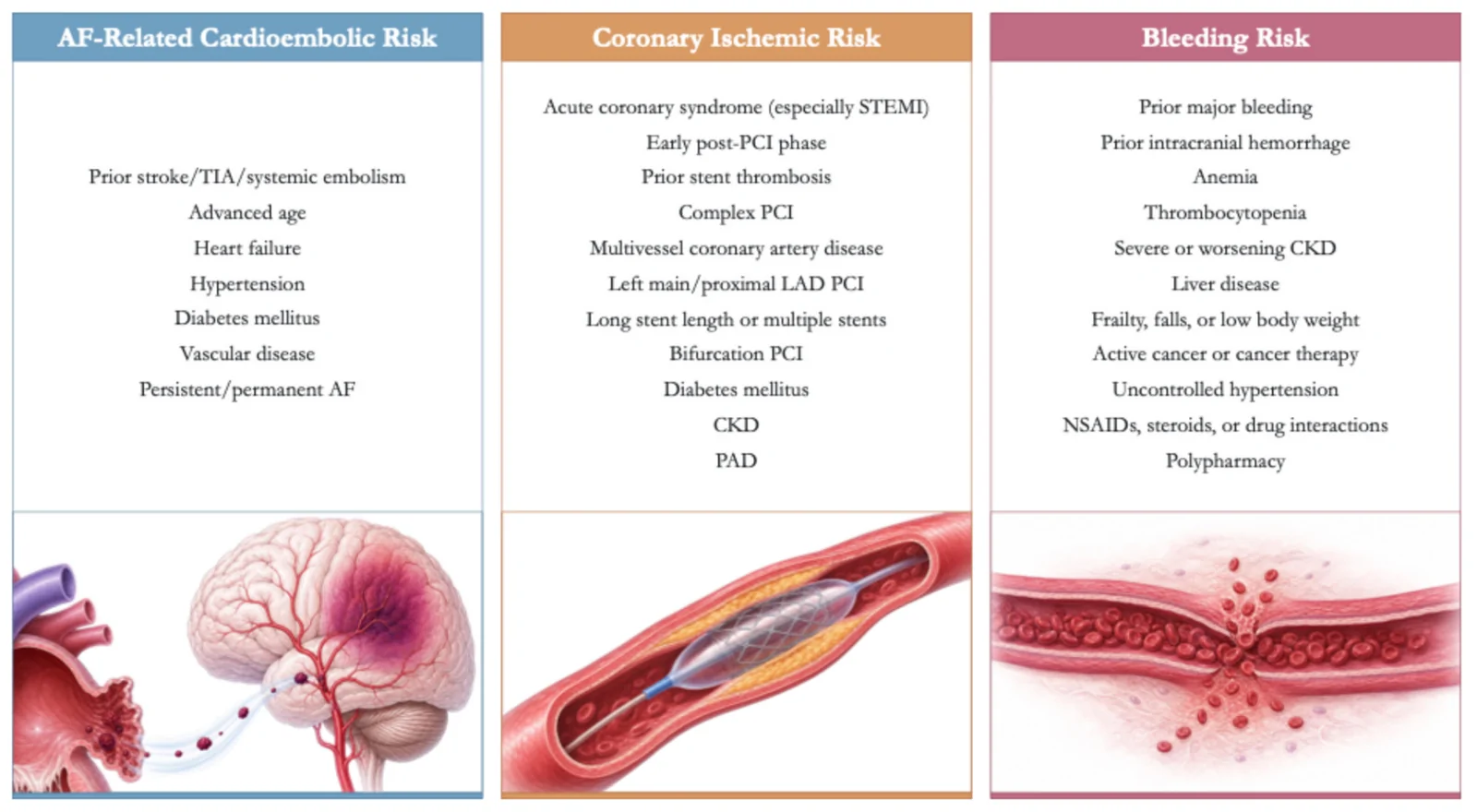
Determinants of Thrombotic and Bleeding Risks in Patients with Atrial Fibrillation and Coronary Artery Disease. Figure summarizing major clinical determinants of thromboembolic, coronary ischemic, and bleeding risk in patients with AF and coronary artery disease. The figure is divided into three vertical panels. The first panel, “AF-related cardioembolic risk,” lists factors associated with thromboembolic risk, including prior stroke, TIA, or systemic embolism; advanced age; heart failure; hypertension; diabetes mellitus; vascular disease; and persistent or permanent AF. The second panel, “Coronary ischemic risk,” lists factors associated with recurrent coronary or stent-related ischemic events, including acute coronary syndrome, especially STEMI; the early post-PCI phase; prior stent thrombosis; complex PCI; multivessel CAD; left main or proximal LAD PCI; long stent length or multiple stents; bifurcation PCI; diabetes mellitus; CKD; and PAD. The third panel, “Bleeding risk,” lists factors associated with hemorrhagic complications, including prior major bleeding; prior intracranial hemorrhage; anemia; thrombocytopenia; severe or worsening CKD; liver disease; frailty, falls, or low body weight; active cancer or cancer therapy; uncontrolled hypertension; NSAID or steroid use, drug interactions; and polypharmacy. Illustrations at the bottom of each panel depict cardioembolism, coronary stenting, and vascular bleeding, respectively. Abbreviations: AF, atrial fibrillation; CAD, coronary artery disease; CKD, chronic kidney disease; LAD, left anterior descending artery; NSAID, nonsteroidal anti-inflammatory drug; PAD, peripheral artery disease; PCI, percutaneous coronary intervention; STEMI, ST-segment elevation myocardial infarction; TIA, transient ischemic attack.

At the same time, AF confers a long-term steady risk of cardioembolism that persists independently of the declining hazard of stent thrombosis [19,29]. Unlike the early-peaking ischemic risk after PCI, the risk of ischemic stroke in AF follows a more linear trajectory over time. Persistent and permanent AF are associated with higher annual stroke rates than paroxysmal presentations, and advancing age together with the accumulation of coexisting conditions—including heart failure and systemic vascular disease—further amplify thromboembolic risk, necessitating periodic reassessment of anticoagulation therapy [29,30]. Bleeding risk during anticoagulation is likewise dynamic and may increase in the presence of prior major bleeding, anemia or thrombocytopenia, renal or hepatic dysfunction, frailty or low body weight, active cancer, uncontrolled hypertension, or concomitant therapies and drug interactions that increase bleeding liability [31,32]. The coexistence of early ischemic vulnerability, sustained cardioembolic risk, and cumulative bleeding hazard defines the central therapeutic challenge in this population (Figure 1). This trade-off is clinically consequential, since ischemic and bleeding events exert comparable prognostic effects [17,33]. An ischemic event identifies a state of persistent vulnerability, with the risk of cardiovascular death, myocardial infarction (MI), or stroke declining from approximately 41% immediately after discharge to about 6% beyond the first year [34]. Similarly, major bleeding after ACS is associated with up to a 5-fold increase in mortality [2]. Based on the classification by the Bleeding Academic Research Consortium, type 3b bleeding carries a prognostic impact comparable to that of MI, whereas type 3c events are associated with even higher mortality [33]. In addition, while late major bleeding is associated with a similar increase in mortality compared to recurrent MI, early major bleeding might have a stronger association with mortality compared to early recurrent MI [35]. Recognition of this prognostic symmetry shifts the therapeutic objective from prevention of thrombosis alone toward maximization of net clinical benefit [17,33,36].

Over the intermediate and long term, progressive attenuation of stent-related ischemic risk in the presence of persistent bleeding hazard provides the rationale for modulation of antithrombotic therapy [37]. Because the relative weight of these competing risks may change over time, treatment intensity and duration should be periodically reassessed and adjusted to the prevailing clinical profile. Once the acute phase has resolved, bleeding risk may exceed residual ischemic risk, supporting de-escalation of treatment intensity without loss of thrombotic protection [17,36]. Shortening DAPT duration, adopting aspirin-free strategies, or switching to less potent P2Y_12_ inhibitors at a certain point in time reflect this evolving balance [38].

## 3. Current Evidence

### 3.1. Establishment of Triple Antithrombotic Therapy and Early VKA-Based Antithrombotic Strategies After PCI

The evolution of antithrombotic strategies in patients with AF undergoing PCI has paralleled advances in both coronary stent technology and OAC [8,39]. During the final decades of the twentieth century, pivotal trials established DAPT as the standard regimen after coronary stent implantation, demonstrating its superiority over OAC alone in preventing stent-related ischemic complications [40,41,42,43]. Conversely, in patients with AF, antiplatelet therapy proved insufficient for the prevention of cardioembolic stroke, whereas OAC consistently demonstrated superior efficacy [44,45].

In clinical practice, this therapeutic dichotomy translated into the empirical adoption of combined antithrombotic regimens incorporating OAC together with antiplatelet therapy in patients with AF undergoing PCI [46]. Before randomized evidence was available, TAT—consisting of a vitamin K antagonist (VKA) plus DAPT—became widely implemented, particularly during the era of early-generation drug-eluting stents, when concerns regarding stent thrombosis were heightened [47]. However, accumulating observational data soon underscored the substantial bleeding burden associated with TAT, raising concerns about the safety and long-term sustainability of this strategy [48,49].

**Figure 2:**
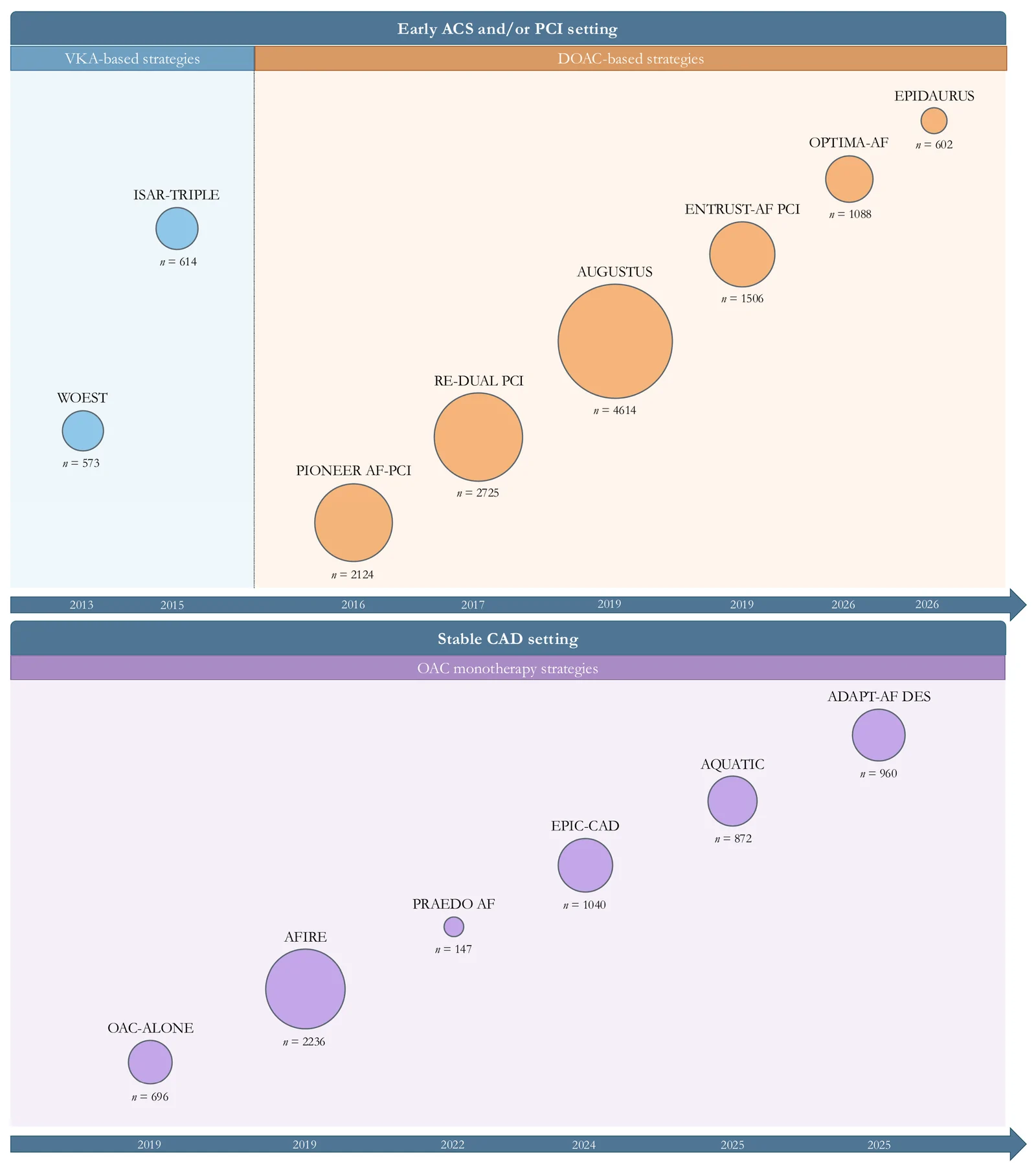
Randomized Trials of Antithrombotic Strategies in Patients with Atrial Fibrillation Undergoing Percutaneous Coronary Intervention. Timeline-based evidence map of randomized trials evaluating antithrombotic strategies in patients with AF across early ACS and/or PCI and stable CAD settings. Trials are displayed in separate panels according to clinical setting and grouped into VKA-based strategies, DOAC-based strategies, and OAC monotherapy strategies. Within each panel, trials are shown chronologically by year of publication, with bubble size proportional to the randomized sample size and bubble fill color identifying the antithrombotic strategy group. The figure illustrates the evolution of randomized evidence from VKA-based combination strategies toward DOAC-based strategies in the early ACS and/or PCI setting and OAC monotherapy strategies in stable CAD. Abbreviations: ACS, acute coronary syndrome; AF, atrial fibrillation; CAD, coronary artery disease; DOAC, direct oral anticoagulant; OAC, oral anticoagulation; PCI, percutaneous coronary intervention; VKA, vitamin K antagonist.

The first randomized efforts to mitigate bleeding risk while preserving antithrombotic efficacy emerged with the WOEST and ISAR-TRIPLE trials (Table 1; Figure 2) [50,51]. In WOEST, which enrolled 573 patients requiring OAC who underwent PCI, dual antithrombotic therapy (DAT) consisting of a VKA plus clopidogrel significantly reduced bleeding complications at 1 year compared with TAT including aspirin (19.4% vs. 44.4%; hazard ratio [HR], 0.36; 95% confidence interval [CI], 0.26–0.50; *p* < 0.0001), without an apparent increase in thrombotic events [50]. Complementing these findings, the ISAR-TRIPLE trial randomized 614 patients receiving OAC after drug-eluting stent implantation to 6 weeks vs. 6 months of TAT, thereby abbreviating clopidogrel exposure [51]. At 9 months, shortening clopidogrel duration was not associated with improved net clinical outcomes compared with prolonged therapy (9.8% vs. 8.8%; HR, 1.14; 95% CI, 0.68–1.91; *p* = 0.63).

Collectively, these early trials challenged the routine use and optimal duration of TAT and provided the conceptual and clinical foundation for subsequent investigations evaluating simplified antithrombotic strategies, particularly those incorporating direct oral anticoagulants (DOACs).

### 3.2. Transition to DOAC-Based Antithrombotic Strategies After PCI

The advent of DOACs has fundamentally reshaped antithrombotic therapy in patients with nonvalvular AF. Large randomized trials have demonstrated the noninferiority—and in selected contexts, superiority—of DOACs as compared to VKAs for the prevention of stroke and systemic embolism, with consistent reductions in major bleeding and more predictable pharmacokinetic profiles [64,65,66,67]. Nevertheless, the concomitant use of antiplatelet therapy confers a synergistic increase in bleeding risk, a burden that escalates in parallel with regimen complexity [68,69]. This persistent imbalance between ischemic protection and hemorrhagic liability provided the rationale for evaluating DOAC-based de-escalation strategies in patients with AF undergoing PCI.

In the PIONEER AF-PCI trial, 2124 patients were randomly assigned to two rivaroxaban-based regimens or to TAT consisting of a VKA plus DAPT [52]. Rivaroxaban administered at 15 mg once daily plus a P2Y_12_ inhibitor or at 2.5 mg twice daily plus DAPT significantly reduced clinically relevant bleeding as compared with VKA-based TAT (16.8% and 18.0% vs. 26.7%; HR, 0.59; 95% CI, 0.47–0.76; *p* < 0.001; and HR, 0.63; 95% CI, 0.50–0.80; *p* < 0.001, respectively), with consistent benefit across strata of procedural and anatomical complexity [70]. The trial was not powered to detect differences in ischemic outcomes, and rivaroxaban doses were lower than those approved for stroke prevention in AF. Similarly, in the RE-DUAL PCI trial, DAT with dabigatran (110 mg or 150 mg twice daily) plus a P2Y_12_ inhibitor (clopidogrel or ticagrelor) was compared with VKA-based TAT including aspirin for 1–3 months [53]. At a mean follow-up of 14 months, DAT significantly reduced major or clinically relevant nonmajor bleeding (HR, 0.52; 95% CI, 0.42–0.63; *p* < 0.001 for superiority with 110 mg; HR, 0.72; 95% CI, 0.58–0.88; *p* < 0.001 for noninferiority with 150 mg), without evidence of excess thromboembolic events. The reduction in bleeding was consistent among patients presenting with ACS—including STEMI—and those undergoing elective PCI [71,72], irrespective of P2Y_12_ inhibitor choice or procedural complexity [71,73]. Subsequent analyses suggested an early net clinical benefit, even among patients at high ischemic and bleeding risk [74].

The AUGUSTUS trial addressed prior methodological limitations through a 2 × 2 factorial design separating anticoagulant and antiplatelet effects [54]. A total of 4614 patients with AF and ACS or PCI were randomly assigned to receive apixaban or a VKA and, independently, aspirin or placebo, in addition to a P2Y_12_ inhibitor for 6 months. Apixaban reduced the primary outcome of major or clinically relevant nonmajor bleeding as compared with VKA therapy (10.5% vs. 14.7%; HR, 0.69; 95% CI, 0.58–0.81; *p* < 0.001), whereas aspirin nearly doubled the risk of bleeding relative to placebo (16.1% vs. 9.0%; HR, 1.89; 95% CI, 1.59–2.24; *p* < 0.001). These findings were consistent irrespective of acute or chronic presentation [75]. Post hoc analyses documented low rates of stent thrombosis at 6 months (1.6%), occurring predominantly within 30 days and being numerically lower with apixaban than with VKA (0.74% vs. 0.97%; HR, 0.76; 95% CI, 0.37–1.56) as well as with aspirin than with placebo (0.63% vs. 1.08%; HR, 0.58; 95% CI, 0.28–1.22); however, it should be noted that aspirin conferred a disproportionate increase in bleeding and that the trial was not powered for definitive ischemic comparisons [76]. Time-dependent analyses indicated that the ischemic benefit of aspirin was confined to the early period, whereas beyond 30 days bleeding risk predominated without a reduction in ischemic events [77]. Overall, apixaban combined with a P2Y_12_ inhibitor without aspirin was associated with improved global outcomes, including reductions in mortality and hospitalizations [78,79]. In the subsequent ENTRUST-AF PCI trial, 1506 patients were assigned to edoxaban-based DAT (60 mg or 30 mg once daily) plus a P2Y_12_ inhibitor or to VKA-based TAT [55]. At 12 months, edoxaban-based DAT was noninferior with respect to major or clinically relevant nonmajor bleeding (17% vs. 20%; HR, 0.83; 95% CI, 0.65–1.05; *p* = 0.001 for noninferiority), with largely overlapping ischemic event rates across acute and chronic presentations [80]. Across these trials, most patients received a short course of TAT before randomization, reflecting contemporary practice.

A relevant residual knowledge gap concerned the choice of P2Y_12_ inhibitor within DOAC-based DAT, as potent P2Y_12_ inhibitors were poorly represented in the landmark randomized trials. The EPIDAURUS trial specifically addressed this uncertainty by comparing, in patients with AF and ACS undergoing PCI, 1 month of DAT with a direct factor Xa inhibitor plus prasugrel or ticagrelor, followed by DOAC plus clopidogrel, versus DOAC plus clopidogrel and in-hospital aspirin, but was prematurely terminated after enrolment of 602 patients due to safety concerns [57]. The prespecified superiority criterion for the primary efficacy endpoint at 6 weeks was not met (win-loss ratio, 1.19; 95% CI, 0.61–2.32; *p* = 0.610), precluding confirmatory interpretation of the hierarchically subsequent primary safety endpoint, while exploratory analyses showed higher rates of Bleeding Academic Research Consortium type ≥2 (27 vs. 16; HR, 1.87; 95% CI, 1.01–3.47) and type ≥3 bleeding (13 vs. 4; HR, 3.54; 95% CI, 1.15–10.85), providing no support for routine use of potent P2Y_12_ inhibition in this setting.

The OPTIMA-AF trial further challenged the need for prolonged combination therapy after PCI in patients with AF. In this multicentre randomized trial, 1088 patients (95% with chronic coronary syndrome [CCS]) undergoing PCI with intravascular imaging guidance were assigned to either 1 month or 12 months of DOAC-based DAT followed by DOAC monotherapy [56]. The abbreviated strategy was noninferior to 12-month DAT for the composite of death or thromboembolic events (5.4% vs. 4.3%; absolute difference, 1.1%; 95% CI, −1.5 to 3.6; HR, 1.25; 95% CI, 0.73–2.17) and significantly reduced major or clinically relevant nonmajor bleeding (4.5% vs. 8.8%; HR, 0.50; 95% CI, 0.30–0.81). These findings provide the first randomized evidence supporting very early transition to DOAC monotherapy in selected patients with AF undergoing PCI, although their applicability remains limited by the exclusion of acute MI and the predominantly lower-risk population treated with intravascular imaging-guided PCI. In addition, findings on efficacy endpoints should be interpreted with caution in light of the lower-than-anticipated event rates [81].

A network meta-analysis incorporating data from five randomized trials and 11,532 patients demonstrated that DOAC-based DAT with a P2Y_12_ inhibitor significantly reduced major bleeding as compared with VKA-based TAT (odds ratio, 0.52; 95% CI, 0.35–0.79), without significant differences in antithrombotic efficacy [82]. A trial sequential analysis confirmed that these findings are likely to be conclusive, both for bleeding and ischemic events [46,83]. The net clinical benefit was consistent across the clinical spectrum, including both ACS and elective PCI (risk ratio [RR], 0.63; 95% CI, 0.56–0.71; *p* < 0.0001; and RR, 0.68; 95% CI, 0.55–0.85; *p* = 0.0008, respectively) [84]. Furthermore, abbreviated DAPT regimens of 6 weeks or less were associated with additional reductions in major or clinically relevant nonmajor bleeding (RR, 0.69; 95% CI, 0.52–0.91; *p* = 0.01), without an increase in ischemic risk, supporting early withdrawal of aspirin to optimize the risk-benefit balance [85]. Consistent with this approach, a dedicated subgroup analysis of the MASTER DAPT trial in patients receiving OAC showed no significant differences in major adverse cardiovascular events or net adverse clinical events (NACE) between an abbreviated triple antithrombotic regimen (mean duration, 33 days) and a more prolonged strategy (mean duration, 96 days) [86]. Notwithstanding these findings, pooled analyses with greater statistical power have identified an increased relative risk of stent thrombosis with DAT (RR, 1.59; 95% CI, 1.01–2.50; *p* = 0.04) and a trend toward more MIs, although there were no differences in mortality, stroke, or major adverse cardiovascular events [87]. Real-world evidence, including data from the prospective WOEST 2 registry, corroborates the favorable bleeding profile of DOAC-based DAT in routine clinical practice [88].

Collectively, these data favor a strategy of minimizing triple therapy to the periprocedural window and individualizing antiplatelet intensity according to thrombotic and bleeding risk. Contemporary management favors early discontinuation of aspirin and preferential use of DOAC-based DAT, balancing the prevention of ischemic events with the reduction of bleeding complications.

### 3.3. Long-Term Antithrombotic Therapy Beyond the Early Post-PCI Phase

Although DOAC-based DAT has substantially improved the safety profile of post-PCI management in patients with AF, the progressive attenuation of stent-related ischemic risk over time has raised uncertainty regarding the need for prolonged antiplatelet therapy. As coronary risk stabilizes beyond the early postprocedural phase, OAC alone may provide adequate protection against thromboembolic events while limiting cumulative exposure to bleeding.

The transition toward OAC monotherapy was first explored in the OAC-ALONE trial. However, the study was terminated prematurely because of slow enrolment, and the results were inconclusive [58]. More definitive evidence emerged from the AFIRE trial, in which 2236 Japanese patients with AF and stable CAD—either medically managed or revascularized more than 1 year earlier—were randomly assigned to rivaroxaban monotherapy or rivaroxaban plus a single antiplatelet agent [59]. Rivaroxaban monotherapy was noninferior with respect to the primary efficacy endpoint, a composite of stroke, systemic embolism, MI, unstable angina requiring revascularization, or death from any cause (4.14% vs. 5.75% per patient-year; HR, 0.72; 95% CI, 0.55–0.95; *p* < 0.001 for noninferiority), and superior with respect to major bleeding (1.62% vs. 2.76% per patient-year; HR, 0.59; 95% CI, 0.39–0.89; *p* = 0.01). The trial was stopped early because of excess mortality in the combination-therapy group. At 24 months, analysis of total events showed a lower cumulative incidence of thrombotic and bleeding outcomes with monotherapy than with combination therapy (12.2% vs. 19.2%; HR, 0.62; 95% CI, 0.48–0.80; *p* < 0.001), with mortality after bleeding exceeding that observed after thrombotic events [89]. Among patients who had undergone coronary stenting, the benefits of monotherapy were consistent with those in the overall trial population [90]. A subsequent post hoc analysis demonstrated superior efficacy (HR, 0.62; 95% CI, 0.45–0.85; *p* = 0.003) and safety (HR, 0.62; 95% CI, 0.39–0.98; *p* = 0.042) of monotherapy among revascularized patients at high thrombotic risk [91].

Additional support for OAC monotherapy has been derived from randomized trials conducted in East Asian populations. In the EPIC-CAD trial, 1040 patients with AF and stable CAD—either medically managed or previously revascularized (≥6 months after CCS or ≥1 year after ACS)—were assigned to edoxaban monotherapy or edoxaban plus a single antiplatelet agent [61]. At 12 months, the primary endpoint of NACE occurred significantly less frequently in the monotherapy group (6.8% vs. 16.2%; HR, 0.44; 95% CI, 0.30–0.65; *p* < 0.001). Similarly, in the ADAPT-AF DES trial, which enrolled 960 patients with AF who had undergone drug-eluting stent implantation at least 1 year before randomization, DOAC monotherapy reduced the incidence of NACE at 12 months as compared with DOAC-based DAT with clopidogrel (9.6% vs. 17.2%; HR, 0.54; 95% CI, 0.37–0.77; *p* < 0.001 for superiority) [63]. In both trials, the observed benefit was driven primarily by reductions in major and clinically relevant nonmajor bleeding [61,63].

Consistent findings were reported in the PRAEDO AF study, which enrolled 147 Japanese patients with nonvalvular AF and stable CAD beyond 6 months after third-generation drug-eluting stent implantation (or ≥1 year after earlier-generation stents) [60]. Edoxaban monotherapy was associated with acceptable clinical safety, with numerically lower bleeding rates than edoxaban plus clopidogrel and no ischemic events in either group [60].

Further insight into the ischemic safety of OAC monotherapy is provided by the AQUATIC trial, conducted in France and involving 872 patients with CCS at high atherothrombotic risk who had undergone stent implantation more than 6 months earlier and required long-term anticoagulation, most commonly for AF [62]. The addition of aspirin to OAC therapy was associated with a significantly higher incidence of the primary composite endpoint—cardiovascular death, MI, stroke, systemic embolism, coronary revascularization, or acute limb ischemia—than with OAC plus placebo (16.9% vs. 12.1%; adjusted HR, 1.53; 95% CI, 1.07–2.18; *p* = 0.02). After a median follow-up of 2.2 years, the trial was terminated because of excess all-cause mortality in the aspirin group, which was also associated with a substantially higher rate of major bleeding (adjusted HR, 3.35; 95% CI, 1.87–6.00; *p* < 0.001) [62].

Taken together, these findings are reinforced by a meta-analysis of four randomized controlled trials including 4092 patients with AF and stable CAD [92]. In the absence of significant differences in ischemic outcomes, OAC monotherapy was associated with a significantly lower risk of bleeding than DOAC-based DAT, supporting a simplified long-term antithrombotic strategy in patients with AF and stable CAD beyond the early post-revascularization period [92].

## 4. Guideline Recommendations

The 2024 European Society of Cardiology (ESC) guidelines and the 2023 American College of Cardiology/American Heart Association/American College of Chest Physicians/Heart Rhythm Society (ACC/AHA/ACCP/HRS) guidelines for the management of AF are largely concordant in their recommendations for antithrombotic therapy in patients with AF undergoing PCI. In both documents, OAC is the cornerstone of treatment in eligible patients, whereas antiplatelet therapy alone is not recommended as an alternative for stroke prevention (class of recommendation [COR] III, level of evidence [LOE] A in the ESC guidelines) [93,94]. When combination therapy is required, DOACs are preferred over VKAs because of their more favorable safety profile (COR I, LOE A in both documents) [93,94]. The ESC guidelines further emphasize that DOAC dose reduction is not recommended in the absence of drug-specific criteria (COR III, LOE B) and that, when a VKA is combined with antiplatelet therapy, an international normalized ratio of 2.0–2.5 with a time in therapeutic range of more than 70% should be maintained (COR IIa, LOE C) [93].

A second area of broad agreement is the avoidance of prolonged TAT as the default strategy. Both the European and North American guidelines recommend that TAT be limited to the periprocedural phase, followed by DAT with OAC and a P2Y_12_ inhibitor, preferably clopidogrel, with early discontinuation of aspirin to reduce bleeding risk [93,94].

In patients with CCS or after uncomplicated elective PCI, ESC guidelines recommend discontinuation of aspirin within 1 week and continuation of DAT for up to 6 months (COR I, LOE A), followed by OAC alone; if ischemic risk clearly outweighs bleeding risk, TAT may be extended up to 1 month (COR IIa) [93,95]. Consistent with this approach, American guidelines recommend OAC alone, rather than OAC plus a single antiplatelet agent, in patients with stable CAD more than 1 year after revascularization (COR I, LOE B–R) [94].

In ACS, where early thrombotic risk is greater, ESC recommends a very short course of TAT, up to 1 week, followed by DAT for up to 12 months (COR I, LOE A); in patients at higher ischemic risk, TAT may be extended up to 1 month (COR IIa) [93,96]. The North American recommendations are conceptually similar but more concise, favoring, in most patients with AF and ACS, discontinuation of aspirin after 1–4 weeks of TAT and continuation of DAT thereafter (COR I, LOE B–R) [94,97].

## 5. Risk Stratification and Antithrombotic Management in Special Populations

Although current guidelines outline a pragmatic approach to antithrombotic therapy in patients with AF undergoing PCI [93], implementation in practice remains challenging. In these patients, AF-related thromboembolic risk, coronary and procedural ischemic risk and bleeding risk often coexist, overlap, and evolve over time [21]. Treatment decisions should therefore integrate the clinical and procedural determinants that influence the balance between ischemic and bleeding risk in the individual patient [98]. In this setting, risk stratification may help tailor the intensity and duration of therapy and thereby improve outcomes [98,99]. However, available tools have only modest predictive performance and often fail to capture the complexity and dynamic nature of risk, which changes with aging, emerging or concomitant conditions, and the clinical course [98,100,101].

For cardioembolic risk, the CHA_2_DS_2_-VA score is the most practical tool for guiding decisions about OAC [93]. Since anticoagulation is generally the default strategy in patients with AF, the main practical value of the score is to identify those at very low risk, in whom treatment may be unnecessary [100,102]. By contrast, ischemic risk after PCI is driven largely by factors that fall outside traditional AF-related scores, including clinical presentation, prior stent thrombosis during antiplatelet therapy, and the anatomic and procedural complexity of the intervention, factors that may inform the intensity and duration of combination antithrombotic therapy [36,96]. For bleeding risk, the HAS-BLED score remains the most useful tool in routine practice for patients with AF [93]. However, a high score (≥3) is not itself a contraindication to OAC; rather, it identifies patients in whom correction of modifiable risk factors and closer follow-up may reduce bleeding events [93,101]. In the PCI setting, the Academic Research Consortium for HBR criteria provide complementary information by capturing determinants beyond the need for anticoagulation alone [36,103]. Scores such as PRECISE-DAPT and PRECISE-HBR may provide additional prognostic information, although their role in guiding treatment decisions in patients with AF undergoing PCI remains less well established [99,104]. Accordingly, treatment decisions should not rely rigidly on individual scores but rather on an integrated qualitative or semiquantitative assessment informed by clinical judgment and patient context [21,36].

These limitations are most apparent in patients with frailty or substantial clinical complexity, in whom the balance between ischemic benefit and bleeding risk often extends beyond what existing scores can capture. Although advanced age, frailty, and polypharmacy increase both ischemic and bleeding risk, they are not in themselves reasons to withhold anticoagulation, which generally retains a favorable net clinical benefit even in this population [93,105,106,107]. At the same time, undertreatment, inappropriate dose reduction, and drug interactions remain common, underscoring the need for multidimensional assessment that includes functional status, fall susceptibility, body weight, renal function, and concomitant therapies [108,109]. Similar considerations apply to chronic kidney disease, which increases both ischemic and bleeding risk and complicates management because DOAC clearance depends on renal function; although OAC generally retains a favorable profile in mild-to-moderate disease, treatment in more advanced stages requires stricter individualization [110,111,112]. Ethnicity may also influence the balance between ischemic protection and bleeding liability; in particular, East Asian patients appear to exhibit the so-called East Asian paradox, characterized by a lower propensity for thrombotic events but a higher susceptibility to bleeding during antithrombotic therapy, suggesting that treatment intensity should be carefully individualized [113]. Further challenges arise in patients with persistently high ischemic risk after PCI, in whom the potential benefit of more intensive or prolonged antithrombotic therapy may be greater, but should still be weighed carefully against the competing risk of bleeding [36]. Management is likewise difficult in patients with previous major bleeding or at very HBR, in whom therapy should focus on correction of modifiable factors and close follow-up, with left atrial appendage occlusion reserved for circumstances in which OAC is truly contraindicated or not feasible [11,93]. Cancer represents another particularly unstable setting, in which anticancer therapies, thrombocytopenia, organ dysfunction, and drug interactions may simultaneously amplify thrombotic and bleeding risk, warranting periodic reassessment of treatment intensity and duration [114].

Overall, these considerations underscore that antithrombotic management in patients with AF undergoing PCI cannot be rigidly standardized. Rather, it demands a dynamic strategy grounded in continuous reassessment of ischemic and bleeding risks, correction of modifiable risk factors, and, in the most complex cases, a multidisciplinary approach.

## 6. Expert Opinion and Future Directions

Current evidence has increasingly shifted antithrombotic therapy management in patients with AF undergoing PCI toward simpler regimens [52,53,54,55]. However, bleeding remains a major concern when combining antithrombotic agents. Randomized trials have consistently shown the bleeding advantage of DAT over TAT, but they were not powered to exclude with similar confidence differences in less frequent coronary ischemic events, particularly stent thrombosis and MI [46]. This evolution has also been supported by the improved safety of newer-generation drug-eluting stents, which has reduced the background risk of stent thrombosis in contemporary practice [36,115]. Thus, the clinically relevant question is no longer whether DAT is preferable to TAT, but which patients can safely undergo very early treatment de-escalation and which may still require more intensive early ischemic protection.

The most contested issue remains the timing of aspirin withdrawal. Most ischemic events after ACS or PCI occur within the first month, precisely when the risk of stent thrombosis is greatest [36]. Since aspirin was used during the periprocedural phase in all major trials, the debate now centers on which patients should continue aspirin beyond this phase and for how long [36,116,117]. In patients at low thrombotic risk or HBR, early aspirin discontinuation with continuation of a DOAC plus clopidogrel appears appropriate. By contrast, in patients with high-risk ACS, prior stent thrombosis, or very complex PCI, a more intensive initial strategy may still be reasonable, provided that bleeding risk is not prohibitive [14,116]. However, beyond the first month, the benefit-risk profile of aspirin becomes substantially less favorable [36]. In this setting, TAT should not be used routinely or continued longer than necessary, though it may remain appropriate in selected patients at high ischemic risk.

**Figure 3:**
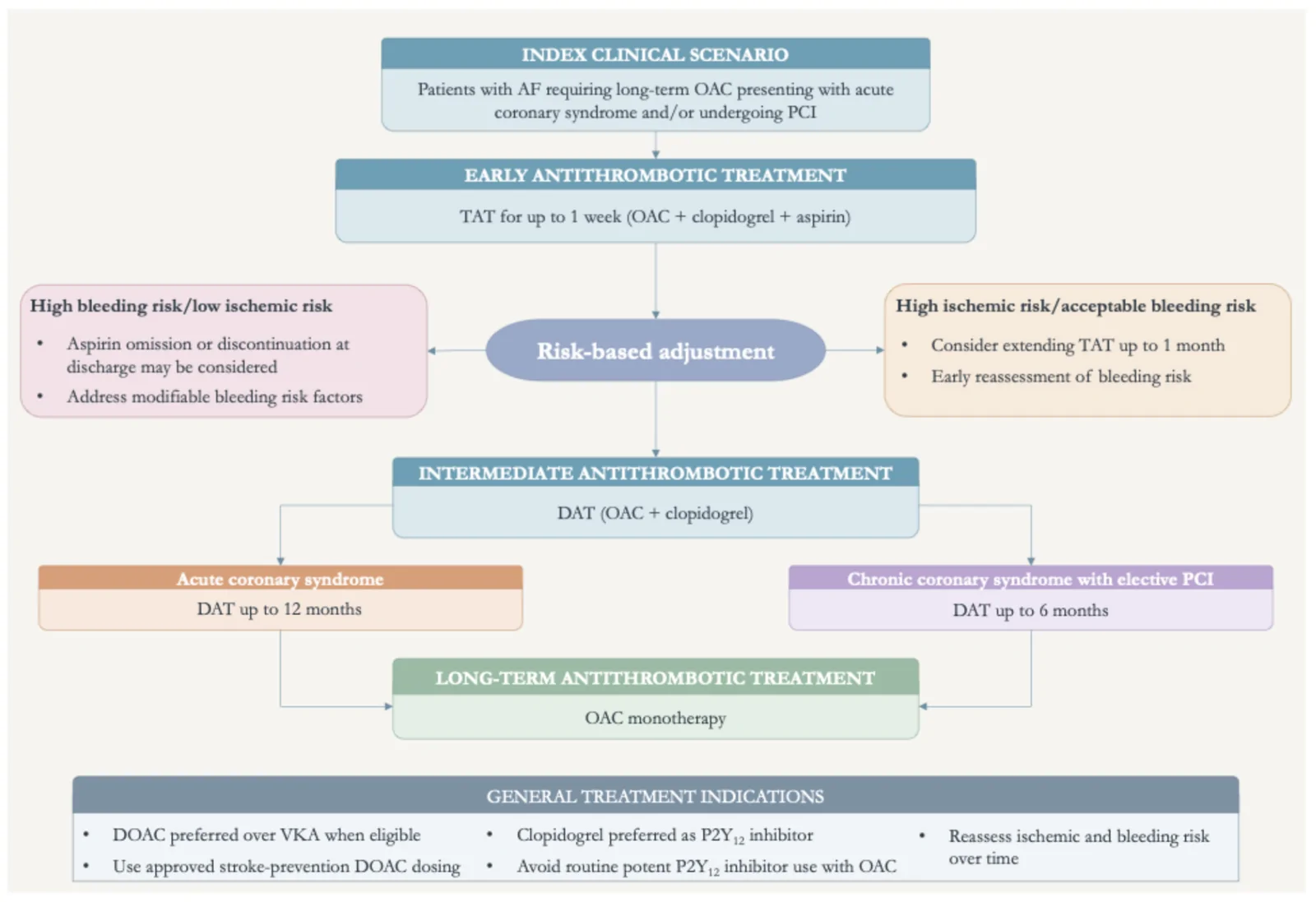
Therapeutic Algorithm for Antithrombotic Therapy Management in Patients with Atrial Fibrillation Undergoing Percutaneous Coronary Intervention. Therapeutic algorithm for phase-specific antithrombotic management in patients with AF requiring long-term OAC who present with ACS and/or undergo PCI. The algorithm begins with an index clinical scenario and recommends early TAT for up to 1 week with OAC, clopidogrel, and aspirin. A central risk-based adjustment step allows treatment modification according to bleeding and ischemic risk. In patients with high bleeding risk and low ischemic risk, aspirin omission or discontinuation at discharge may be considered, together with correction of modifiable bleeding risk factors. In patients with high ischemic risk and acceptable bleeding risk, extension of TAT up to 1 month may be considered, with early reassessment of bleeding risk. The intermediate phase consists of DAT with OAC plus clopidogrel. Duration is differentiated by clinical setting: up to 12 months after ACS and up to 6 months in patients with CCS undergoing elective PCI. Both pathways transition to long-term OAC monotherapy. General treatment principles include preferring DOACs over VKAs when eligible, using approved stroke-prevention DOAC dosing, preferring clopidogrel as the P2Y_12_ inhibitor, avoiding routine use of potent P2Y_12_ inhibitors with OAC, and reassessing ischemic and bleeding risk over time. The algorithm is aligned with contemporary guideline recommendations and provides a practical, phase-specific representation of how combination therapy may be shortened or extended according to the evolving balance between ischemic and bleeding risk. Abbreviations: ACS, acute coronary syndrome; AF, atrial fibrillation; CCS, chronic coronary syndrome; DAT, dual antithrombotic therapy; DOAC, direct oral anticoagulant; OAC, oral anticoagulation; PCI, percutaneous coronary intervention; TAT, triple antithrombotic therapy; VKA, vitamin K antagonist.

Conversely, bleeding risk should not automatically lead to undertreatment. A substantial proportion of patients with AF undergoing PCI represent a bi-risk population, in whom ischemic and bleeding vulnerability coexist [17,118]. In such patients, reflex simplification and indiscriminate intensification can be equally inappropriate, particularly when the DOAC dose is reduced without a clear indication in response to minor bleeding. More broadly, antithrombotic therapy after PCI should be viewed not as a fixed decision made at the time of the procedure, but as a dynamic, phase-specific process in which risk scores may inform, but should not replace, clinical judgment (Figure 3) [14,119].

In light of these considerations, future research should focus less on the regimen itself than on identifying the patients in whom ischemic protection should take precedence over bleeding avoidance, or vice versa. Although results in patients with AF are controversial, factor XI inhibitors may be a promising option due to their mechanistic rationale [120,121]. The central unmet need is to reduce bleeding without sacrificing antithrombotic efficacy, especially when anticoagulants are combined with antiplatelet therapy [122].

Several ongoing trials are expected to refine the optimal intensity and duration of combination therapy in patients with AF undergoing PCI (Table 2). WOEST 3 (NCT04436978) is testing a 1-month course of DAPT followed by DAT, as compared with contemporary standard care. Other studies are focusing on patients with ACS, in whom early ischemic risk is greatest and the role of more intensive platelet inhibition remains uncertain. OPTIMA-4 (NCT03234114) is evaluating 12 months of DAT with dabigatran 110 mg twice daily combined with either ticagrelor or clopidogrel. ADONIS-PCI (NCT04695106) is testing dabigatran plus ticagrelor, with ticagrelor de-escalation after the first month, as compared with standard care. Finally, MATRIX-2 (NCT05955365) is exploring a more radical simplification strategy, comparing 1-month P2Y_12_ inhibitor monotherapy followed by OAC alone with current standard care.

## 7. Conclusions

In patients with AF undergoing PCI, antithrombotic therapy requires balancing protection from cardioembolism and coronary ischemic events against bleeding. Randomized evidence has shifted practice away from prolonged TAT toward DOAC-based DAT with early aspirin discontinuation, improving safety. Treatment intensity and duration should reflect clinical presentation, procedural complexity, coexisting conditions, and changes in risk over time. In practice, aspirin should generally be stopped at discharge or within 1 week, with DOAC plus clopidogrel continued for up to 12 months after ACS and up to 6 months after elective PCI; TAT may be extended to 1 month when ischemic risk is high and bleeding risk acceptable. Clopidogrel remains the preferred P2Y_12_ inhibitor with OAC, as routine prasugrel or ticagrelor use is not supported. In selected patients with CCS undergoing PCI, further shortening the DAT phase and transitioning to OAC monotherapy as early as 1 month may be considered, while OAC alone remains the preferred long-term strategy. Controversy persists over how early therapy can be simplified, which patients need stronger early ischemic protection, and when OAC monotherapy should begin. Future research should clarify these remaining uncertainties through more precise patient selection and strategies that further reduce bleeding without compromising ischemic efficacy.

## Figures and Tables

**Table 1:** Landmark Randomized Trials of Antithrombotic Strategies in Patients Requiring Oral Anticoagulation Undergoing Percutaneous Coronary Intervention.

Trial (Year)	Population (*n*)	Intervention	Comparator	Primary Endpoint(s)	Main Results
VKA-based strategies					
WOEST [50] (2013)	Patients on long-term OAC undergoing PCI (69% AF/flutter) (*n* = 573)	VKA plus SAPT (clopidogrel)	VKA plus DAPT (clopidogrel + aspirin)	Any bleeding at 1 year	19.4% vs. 44.4% (HR, 0.36; 95% CI, 0.26–0.50; *p* < 0.0001)
ISAR-TRIPLE [51] (2015)	Patients on long-term OAC undergoing PCI with DES implantation (84% AF/flutter) (*n* = 614)	6-week TAT^a^, then VKA plus SAPT (aspirin)	6-month TAT^a^	Death, MI, definite ST, stroke, or TIMI major bleeding at 9 months	9.8% vs. 8.8% (HR, 1.14; 95% CI, 0.68–1.91; *p* = 0.63)
DOAC-based strategies					
PIONEER AF-PCI [52] (2016)	Patients with NVAF undergoing PCI with stent implantation (*n* = 2124)	Rivaroxaban 15 mg once daily plus SAPT (P2Y_12_i), or rivaroxaban 2.5 mg twice daily plus DAPT (P2Y_12_i + aspirin)	VKA plus DAPT (P2Y_12_i + aspirin)	Clinically significant bleeding^b^ at 12 months	16.8% and 18.0% vs. 26.7% (HR, 0.59; 95% CI, 0.47–0.76; *p* < 0.001; and HR, 0.63; 95% CI, 0.50–0.80; *p* < 0.001)
RE-DUAL PCI [53] (2017)	Patients with NVAF undergoing PCI with stent implantation (*n* = 2725)	Dabigatran 110 mg or 150 mg twice daily plus SAPT (P2Y_12_i)	Warfarin plus DAPT (P2Y_12_i + aspirin)	ISTH major or CRNM bleeding over a mean of 14 months	15.4% vs. 26.9% (HR, 0.52; 95% CI, 0.42–0.63; *p* < 0.001 for both noninferiority and superiority) and 20.2% vs. 25.7% (HR, 0.72; 95% CI, 0.58–0.88; *p* < 0.001 for noninferiority; *p* = 0.002 for superiority)
AUGUSTUS [54] (2019)	Patients with AF and recent ACS and/or PCI planned for P2Y_12_i therapy (*n* = 4614)	Apixaban plus P2Y_12_i; placebo plus P2Y_12_i	VKA plus P2Y_12_i; aspirin plus P2Y_12_i	ISTH major or CRNM bleeding at 6 months	- Apixaban vs. VKA: 10.5% vs. 14.7% (HR, 0.69; 95% CI, 0.58–0.81; *p* < 0.001 for both noninferiority and superiority); - Aspirin vs. placebo: 16.1% vs. 9.0% (HR, 1.89; 95% CI, 1.59–2.24; *p* < 0.001)
ENTRUST-AF PCI [55] (2019)	Patients with NVAF undergoing PCI for stable CAD or ACS (*n* = 1506)	Edoxaban plus SAPT (P2Y_12_i)	VKA plus DAPT (P2Y_12_i + aspirin)	ISTH major or CRNM bleeding at 12 months	17% vs. 20% (HR, 0.83; 95% CI, 0.65–1.05; *p* = 0.0010 for noninferiority; *p* = 0.1154 for superiority)
OPTIMA-AF [56] (2026)	Patients with AF undergoing PCI with stent implantation (*n* = 1088)	1-month DOAC plus SAPT (P2Y_12_i), then DOAC alone	12-month DOAC plus SAPT (P2Y_12_i), then DOAC alone	- Efficacy: death or thromboembolic events at 1 year; - Safety: ISTH major or CRNM bleeding at 1 year	- Efficacy: 5.4% vs. 4.3% (absolute difference, 1.1%; 95% CI, −1.5 to 3.6; HR, 1.25; 95% CI, 0.73–2.17; noninferior); - Safety: 4.5% vs. 8.8% (absolute difference, −4.4%; 95% CI, −7.3 to −1.4; HR, 0.50; 95% CI, 0.30–0.81; *p* = 0.0041 for superiority)
EPIDAURUS [57] (2026)	Patients with AF and ACS undergoing PCI (*n* = 602 of 1474 planned)	1-month DOAC^c^ plus a potent P2Y_12_i, then DOAC^c^ plus SAPT (clopidogrel)	DOAC^c^ plus clopidogrel and in-hospital aspirin	- Efficacy (hierarchical): death, ST, MI, ischemic stroke, SE, or urgent revascularization at 6 weeks; - Safety (hierarchical): death or BARC type 2–3 bleeding at 6 weeks	- Efficacy: win-loss ratio, 1.19 (95% CI, 0.61–2.32; *p* = 0.610 for superiority). - Safety: win-loss ratio, 0.66 (95% CI, 0.39–1.11; *p* = 0.713 for noninferiority)^d^
OAC-based long-term strategies					
OAC-ALONE [58] (2019)	Patients with AF and stable CAD beyond 1 year after coronary stenting (*n* = 696 of 2000 planned)	OAC alone^e^	OAC^e^ plus SAPT (aspirin or clopidogrel)	Death, MI, stroke, or SE over a median of 2.5 years	15.7% vs. 13.6% (HR, 1.16; 95% CI, 0.79–1.72; *p* = 0.20 for noninferiority; *p* = 0.45 for superiority)
AFIRE [59] (2019)	Patients with AF and stable CAD beyond 1 year after PCI/CABG or with angiographically confirmed CAD not requiring revascularization (*n* = 2236)	Rivaroxaban 15 mg once daily^f^	Rivaroxaban 15 mg once daily^f^ plus SAPT (aspirin or P2Y_12_i)	- Efficacy: stroke, SE, MI, UA requiring revascularization, or death over a median of 24.1 months; - Safety: ISTH major bleeding over a median of 24.1 months	- Efficacy: 4.14% vs. 5.75% per PY (HR, 0.72; 95% CI, 0.55–0.95; *p* < 0.001 for noninferiority); - Safety: 1.62% vs. 2.76% per PY (HR, 0.59; 95% CI, 0.39–0.89; *p* = 0.01 for superiority)
PRAEDO AF [60] (2022)	Patients with NVAF and stable CAD, including ≥6 months after third-generation DES implantation or ≥1 year after other stents (*n* = 147)	Edoxaban alone	Edoxaban plus SAPT (clopidogrel)	ISTH major or clinically significant bleeding over a median of 1.7 years	1.67% vs. 4.28% per PY (HR, 0.39; 95% CI, 0.08–2.02; *p* = 0.26)
EPIC-CAD [61] (2024)	Patients with AF and stable CAD previously treated with revascularization or managed medically (*n* = 1040)	Edoxaban alone	Edoxaban plus SAPT (aspirin or P2Y_12_i)	Death, MI, stroke, SE, unplanned urgent revascularization, or ISTH major or CRNM bleeding at 12 months	6.8% vs. 16.2% (HR, 0.44; 95% CI, 0.30–0.65; *p* < 0.001)
AQUATIC [62] (2025)	Patients with CCS, previous coronary stent implantation, high atherothrombotic risk, receiving long-term OAC (89% AF) (*n* = 872 of 2000 planned)	OAC^g^ plus aspirin	OAC^g^ plus placebo	- Efficacy: CV death, MI, stroke, SE, coronary revascularization, or acute limb ischemia over a median of 2.2 years; - Safety (key): ISTH major bleeding over a median of 2.2 years	- Efficacy: 16.9% vs. 12.1% (adjusted HR, 1.53; 95% CI, 1.07–2.18; *p* = 0.02); - Safety: 10.2% vs. 3.4% (adjusted HR, 3.35; 95% CI, 1.87–6.00; *p* < 0.001)
ADAPT-AF DES [63] (2025)	Patients with AF ≥1 year after second- or third-generation DES implantation (*n* = 960)	DOAC alone	DOAC plus SAPT (clopidogrel)	Death, MI, ST, stroke, SE, or ISTH major or CRNM bleeding at 12 months	9.6% vs. 17.2% (HR, 0.54; 95.2% CI, 0.37–0.77; *p* < 0.001 for both noninferiority and superiority)

^a^TAT consisted of VKA-based OAC plus DAPT with aspirin and clopidogrel. ^b^Clinically significant bleeding was defined as a composite of TIMI major bleeding or minor bleeding, or bleeding requiring medical attention. ^c^Among DOACs, only factor Xa inhibitors (apixaban, edoxaban, or rivaroxaban) were permitted in EPIDAURUS; patients receiving dabigatran or a VKA were required to switch to a factor Xa inhibitor. ^d^Confirmatory interpretation of the primary safety endpoint was precluded because the preceding superiority criterion for the primary efficacy endpoint was not met. ^e^OAC consisted of warfarin in 75.2% and a DOAC in 24.8% of patients. ^f^Rivaroxaban was dose-reduced to 10 mg once daily according to renal function. ^g^OAC consisted of a DOAC in 89.7% and a VKA in 10.3% of patients. Abbreviations: ACS, acute coronary syndrome; AF, atrial fibrillation; BARC, Bleeding Academic Research Consortium; CABG, coronary artery bypass grafting; CAD, coronary artery disease; CCS, chronic coronary syndrome; CI, confidence interval; CRNM, clinically relevant nonmajor; CV, cardiovascular; DAPT, dual antiplatelet therapy; DES, drug-eluting stent; DOAC, direct oral anticoagulant; HR, hazard ratio; ISTH, International Society on Thrombosis and Haemostasis; MI, myocardial infarction; NVAF, nonvalvular atrial fibrillation; OAC, oral anticoagulation; P2Y_12_i, P2Y_12_ inhibitor; PCI, percutaneous coronary intervention; PY, patient-year; SAPT, single antiplatelet therapy; SE, systemic embolism; ST, stent thrombosis; TAT, triple antithrombotic therapy; TIMI, Thrombolysis In Myocardial Infarction; UA, unstable angina; VKA, vitamin K antagonist.

**Table 2:** Ongoing Randomized Trials of Antithrombotic Strategies for Patients with Atrial Fibrillation Undergoing Percutaneous Coronary Intervention.

Trial (NCT)	Population	Sample Size (Estimated)	Investigated Strategy	Primary Endpoint(s)	Expected Completion
OPTIMA-4 (NCT03234114)	Patients with NVAF and ACS undergoing PCI with new-generation DES implantation	1472	Dabigatran 110 mg twice daily plus SAPT (ticagrelor) vs. dabigatran 110 mg twice daily plus SAPT (clopidogrel)	- Efficacy: CV death, MI, ischemic stroke, SE, or unplanned revascularization at 12 months; - Safety: ISTH major bleeding or CRNM bleeding at 12 months	NA
ADONIS-PCI (NCT04695106)	Patients with NVAF and ACS undergoing PCI with stent implantation	1194	Dabigatran plus SAPT (ticagrelor) vs. dabigatran plus DAPT (aspirin + clopidogrel)	ISTH major or CRNM bleeding at 24 months	2026
WOEST 3 (NCT04436978)	Patients with AF/flutter undergoing PCI	2000	30-day DAPT (aspirin + P2Y_12_i), then edoxaban plus SAPT (P2Y_12_i) vs. edoxaban plus SAPT (P2Y_12_i), with aspirin for up to 30 days	- Efficacy: death, MI, stroke, SE, or ST at 6 weeks; - Safety: ISTH major bleeding or CRNM bleeding at 6 weeks	2027
MATRIX-2 (NCT05955365)	Patients with AF/flutter undergoing PCI with stent implantation	3010	1-month SAPT (P2Y_12_i), then DOAC alone vs. TAT up to 1 month, then DOAC plus SAPT (P2Y_12_i) for 6–12 months, then DOAC alone	- Efficacy: death, MI, stroke, or non-central nervous system SE at 12 months; - Safety: ISTH major bleeding or CRNM bleeding at 12 months	2028

Abbreviations: ACS, acute coronary syndrome; AF, atrial fibrillation; CRNM, clinically relevant nonmajor; CV, cardiovascular; DAPT, dual antiplatelet therapy; DES, drug-eluting stent; DOAC, direct oral anticoagulant; ISTH, International Society on Thrombosis and Haemostasis; MI, myocardial infarction; NA, not available; NCT, National Clinical Trial identifier; NVAF, nonvalvular atrial fibrillation; P2Y_12_i, P2Y_12_ inhibitor; PCI, percutaneous coronary intervention; SAPT, single antiplatelet therapy; SE, systemic embolism; ST, stent thrombosis; TAT, triple antithrombotic therapy.

## Data Availability

No new data were created or analyzed in this study. Data sharing is not applicable to this article.

## Abbreviations

ACS	acute coronary syndrome
AF	atrial fibrillation
CAD	coronary artery disease
CCS	chronic coronary syndrome
CI	confidence interval
COR	class of recommendation
DAPT	dual antiplatelet therapy
DAT	dual antithrombotic therapy
DOAC	direct oral anticoagulant
ESC	European Society of Cardiology
HBR	high bleeding risk
HR	hazard ratio
LOE	level of evidence
MI	myocardial infarction
NACE	net adverse clinical events
OAC	oral anticoagulation
PCI	percutaneous coronary intervention
RR	risk ratio
STEMI	ST-segment elevation myocardial infarction
TAT	triple antithrombotic therapy
VKA	vitamin K antagonist
